# Role of IL-33 in inflammation and disease

**DOI:** 10.1186/1476-9255-8-22

**Published:** 2011-08-26

**Authors:** Ashley M Miller

**Affiliations:** 1 Institute of Infection, Immunity and Inflammation, College of Medical, Veterinary and Life Sciences, GBRC, University of Glasgow, Glasgow G12 8TA, UK

## Abstract

Interleukin (IL)-33 is a new member of the IL-1 superfamily of cytokines that is expressed by mainly stromal cells, such as epithelial and endothelial cells, and its expression is upregulated following pro-inflammatory stimulation. IL-33 can function both as a traditional cytokine and as a nuclear factor regulating gene transcription. It is thought to function as an 'alarmin' released following cell necrosis to alerting the immune system to tissue damage or stress. It mediates its biological effects via interaction with the receptors ST2 (IL-1RL1) and IL-1 receptor accessory protein (IL-1RAcP), both of which are widely expressed, particularly by innate immune cells and T helper 2 (Th2) cells. IL-33 strongly induces Th2 cytokine production from these cells and can promote the pathogenesis of Th2-related disease such as asthma, atopic dermatitis and anaphylaxis. However, IL-33 has shown various protective effects in cardiovascular diseases such as atherosclerosis, obesity, type 2 diabetes and cardiac remodeling. Thus, the effects of IL-33 are either pro- or anti-inflammatory depending on the disease and the model. In this review the role of IL-33 in the inflammation of several disease pathologies will be discussed, with particular emphasis on recent advances.

## Review

### Basic Biology of IL-33

**Figure 1:**
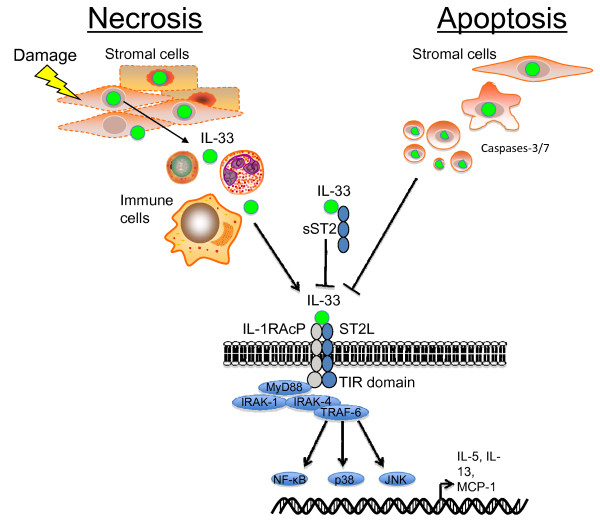
**IL-33 release and signaling via ST2L**. IL-33 is predominantly expressed by stromal cells such as epithelial and endothelial cells. Damage to these cells can induce necrosis and release of full length IL-33 which can activate the heterodimeric ST2L/IL-1RAcP receptor complex on a variety of immune cells or be neutralized by binding to sST2. During apoptosis IL-33 is cleaved by caspases-3/7 leading to its inactivation. Upon activation of ST2L MyD88 and IRAK-1/4 are recruited and this leads to activation of the transcription factor nuclear factor-κB (NF-κB) and the mitogen-activated protein kinase (MAPK) pathway, which is mediated by the activation of the MAPKs extracellular signal-regulated kinase (ERK), p38 and JUN N-terminal kinase (JNK) and ultimately to the production of Th2 cytokines and chemokines.

Interleukin (IL)-33 (also known as IL-1F11) was originally identified as DVS27, a gene up-regulated in canine cerebral vasospasm [1], and as "nuclear factor from high endothelial venules" (NF-HEV) [2]. However, in 2005 analysis of computational structural databases revealed that this protein had close amino acid homology to IL-18, and a β-sheet trefoil fold structure characteristic of IL-1 family members [3]. IL-33 binds to a ST2L (also known as T1, IL-1RL1, DER4), which is a member of the Toll-like receptor (TLR)/IL1R superfamily. IL-33/ST2L then forms a complex with the ubiquitously expressed IL-1R accessory protein (IL-1RAcP) [4-6]. Signaling is induced through the cytoplasmic Toll-interleukin-1 receptor (TIR) domain of IL-1RAcP. This leads to recruitment of the adaptor protein MyD88 and activation of transcription factors such as NF-κB via TRAF6, IRAK-1/4 and MAP kinases and the production of inflammatory mediators (Figure 1) [3]. The ST2 gene can also encode at least 2 other isoforms in addition to ST2L by alternative splicing, including a secreted soluble ST2 (sST2) form which can serve as a decoy receptor for IL-33 [7], and an ST2V variant form present mainly in the gut of humans [8]. Signaling through ST2L also appears to be negatively regulated by the molecule single Ig IL-1R-related molecule (SIGIRR) and IL-33 induced immune responses were enhanced in SIGIRR^-/- ^mice [9].

IL-33 appears to be a cytokine with dual function, acting both as a traditional cytokine through activation of the ST2L receptor complex and as an intracellular nuclear factor with transcriptional regulatory properties [10]. The amino terminus of the IL-33 molecule contains a nuclear localization signal and a homeodomain (helix-turn-helix-like motif) that can bind to heterochromatin in the nucleus and has similar structure to the *Drosophila *transcription factor *engrailed *[2,11]. In a similar manner to which a motif found in Kaposi sarcoma herpesvirus LANA (latency-associated nuclear antigen) attaches its viral genomes to mitotic chromosomes, nuclear IL-33 is thought to be involved in transcriptional repression by binding to the H2A-H2B acidic pocket of nucleosomes and regulating chromatin compaction by promoting nucleosome-nucleosome interactions [12]. However, the specific transcriptional targets or the biological effects of nuclear IL-33 are unclear at present.

Both IL-1β and IL-18 are synthesized as a biologically inactive precursors and activated by caspase-1 cleavage under pro-inflammatory conditions and it was initially thought that IL-33 underwent similar processing by caspase-1 [3]. However, recent studies suggest that proteolytic processing is not required for IL-33 signaling via ST2L [13]. Furthermore, it has been suggested that a new splice variant of IL-33 exists, which lacks the putative caspase-1 cleavage site, and is biologically active inducing signaling via ST2L [14]. In fact, cleavage of IL-33 by caspases appears to mediate inactivation of IL-33 and its pro-inflammatory properties [13,15-17]. Currently, it is thought that full length biologically active IL-33 may be released during necrosis as a endogenous danger signal or 'alarmin', but during apoptosis IL-33 is cleaved by caspases leading to inactivation of its pro-inflammatory properties [18].

### IL-33, an inducer of Th2 immune responses

Unlike the other IL-1 family members IL-33 primarily induces T helper 2 (Th2) immune responses in a number of immune cell types (reviewed in detail in [19]). ST2L was initially shown to be selectively expressed on Th2, but not Th1 [20,21] or regulatory (Treg) T cells [22]. Subsequent studies have shown that IL-33 can activate murine dendritic cells directly driving polarization of naïve T cells towards a Th2 phenotype [23], and it can act directly on Th2 cells to increase secretion of Th2 cytokines such as IL-5 and IL-13 [3,24]. Furthermore, IL-33 can also act as a chemo-attractant for Th2 cells [25]. IL-33 can activate B1 B cells *in vivo*, markedly enhancing production of IgM antibodies and IL-5 and IL-13 production from these cells [3,26,27].

IL-33 is also a potent activator of the innate immune system. Schmitz and co-workers demonstrated that injection of IL-33 into mice induces a profound eosinophilia [3], and has potent effects on this cell type, including induction of superoxide anion and IL-8 production, degranulation and cell survival [28]. Subsequently, it has been shown that IL-33 is also a potent activator of mast cells and basophils and can induce degranulation, maturation, promote survival and the production of several pro-inflammatory cytokines in these cells [29-32]. In neutrophils, IL-33 prevents the down-regulation of CXCR2 and inhibition of chemotaxis induced by the activation of TLR4 [33]. Macrophages constitutively express ST2L and IL-33 can amplify an IL-13-driven polarization of macrophages towards an alternatively activated or M2 phenotype, thus enhancing Th2 immune responses [34]. IL-33 can also enhance LPS-induced production of TNFα in these cells [35].

It is likely that the primary role of these IL-33 effects on the immune system in evolutionary terms was in host defense against pathogens. In fact, IL-33/ST2 have been shown to be highly expressed and protective several parasite infections in animal models in which Th2 cells are host protective, including *Leishmania major *[36,37], *Toxoplasma gondii *[38], *Trichuris muris *[39], and *Nippostrongylus brasiliensis *[40]. Furthermore, a recent discovery has highlighted a new population of cells named nuocytes which expand in response to IL-33 and represent the predominant early source of IL-13 during helminth infection with *Nippostrongylus brasiliensis *[41]. However, it is clear that the potent activatory effects of IL-33 on several immune cell types is likely to impact on various inflammatory diseases.

### Role of the IL-33/ST2 pathway in inflammatory diseases

#### Asthma

Asthma is a chronic inflammatory disease classically characterized by airway hyper-responsiveness, allergic inflammation, elevated serum IgE levels, and increased Th2 cytokine production. Given that IL-33 is a strong inducer of Th2 immune responses its role in asthma has been extensively studied (reviewed in [42]). Initial gene expression studies in a range of tissues using human and mouse cDNA libraries revealed expression of IL-33 in lung tissue, and high expression in bronchial smooth muscle cells [3]. More recently, expression of IL-33 was found in higher levels in endobronchial biopsies from human asthmatic subjects compared to controls. The IL-33 expression was particularly evident in those with severe asthma [43], and the expression was mainly located in bronchial epithelial cells [44]. Studies to investigate which cells were the main IL-33 responsive cells in lung demonstrated that both epithelial and endothelial cells, but not smooth muscle cells or fibroblasts were important [45]. Several animal model studies have highlighted a functionally important role for IL-33/ST2 in asthma and allergic airways inflammation. In a murine ovalbumin-induced airway inflammation model, intranasal administration of IL-33 induces antigen-specific IL-5^+ ^T cells and promotes allergic airway disease even in the absence of IL-4 [24]. Furthermore, intranasal IL-33 also promotes airways hyper-responsiveness, goblet cell hyperplasia, eosinophilia, polarization of macrophages towards an M2 phenotype, and accumulation of lung IL-4, IL-5 and IL-13 [34,46,47]. More recently, an IL-33 transgenic mouse was generated in which IL-33 expression was controlled under a CMV promoter and released as a cleaved 18 kDa protein in pulmonary tissue [48]. These mice developed massive airway inflammation with infiltration of eosinophils, hyperplasia of goblet cells and accumulation of pro-inflammatory cytokines in bronchoalveolar lavage fluid. In contrast, intraperitoneal anti-IL-33 antibody treatment inhibited allergen-induced lung eosinophilic inflammation and mucus hypersecretion in a murine model [49]. Furthermore, administration of blocking anti-ST2 antibodies or ST2-Ig fusion protein inhibited Th2 cytokine production *in vivo*, eosinophilic pulmonary inflammation and airways hyper-responsiveness [50]. At present, the role of IL-33/ST2 in studies using ST2-deficient mice is unclear as these mice are not protected in the ovalbumin-induced airway inflammation model but have attenuated inflammation in a short-term priming model of asthma. Furthermore, there is also an exacerbation of disease in wild-type or Rag-1^-/- ^mice that had undergone adoptive transfer of ST2^-/- ^DO11.10 Th2 cells [24,51,52]. In order to clarify the role of IL-33/ST2 in lung inflammation, several groups have generated mice deficient in IL-33. Oboki and co-workers demonstrated that 2 sensitizations of IL-33^-/- ^mice with ovalbumin emulsified in alum showed attenuated eosinophil and lymphocyte recruitment to the lung, airway hyper-responsiveness and inflammation [19]. A similar study by Louten and colleagues has also shown that endogenous IL-33 contributes to airway inflammation and peripheral antigen-specific responses in ovalbumin-induced acute allergic lung inflammation using IL-33^-/- ^mice [53]. Collectively, the data suggest that IL-33 is involved in lung inflammation and supports the concept of ST2 as a therapeutic target in asthma.

#### Rheumatological diseases

Recent evidence suggests a role for IL-33/ST2 in several rheumatological diseases, including rheumatoid arthritis (RA), osteoarthritis (OA), psoriatic arthritis (PsA) and systemic lupus erythematosus (SLE). The first study to link IL-33 expression with arthritis utilized *in situ *hybridization to show that IL-33 mRNA expression in the RA synovium is primarily in endothelial cells [11]. Subsequently, IL-33 protein has been found in endothelial cells of synovial tissue and in cells morphologically consistent with synovial fibroblasts in a subset of RA, PsA and OA patients [54]. IL-33 is also expressed in cultured synovial fibroblasts from patients with RA and expression was markedly elevated *in vitro *by inflammatory cytokines [55,56]. Circulating IL-33 protein has also been detected in 94/223 RA patient serum samples by ELISA, but was completely absent in healthy controls or OA samples [57]. Furthermore, the level of serum IL-33 decreased after anti-TNF treatment and correlated with production of IgM and RA-related autoantibodies including Rheumatoid Factor and anti-citrullinated protein antibodies. Serum and synovial fluid levels of IL-33 have also been shown to decrease in patients who respond to anti-TNF treatment, while they did not change in non-responders [58]. Similarly, Talabot-Ayer and co-workers show that serum and synovial fluid IL-33 levels were higher in RA than in OA patients, and undetectable in PsA serum and synovial fluid [54]. Another study has demonstrated that neutrophils from patients with RA successfully treated with anti-TNF treatment expressed significantly lower levels of ST2 than patients treated with methotrexate alone [59]. In SLE, one study has shown serum IL-33 levels were significantly increased, compared with healthy controls, but to a lower extent than in patients with RA [60]. The other study reported no change in serum IL-33 levels between controls and SLE patients, but did report a significant increase in sST2 that correlated with SLE disease activity [61].

In murine models of RA, IL-33 mRNA has also been detected in the joints of mice undergoing collagen-induced arthritis (CIA) [56], and in mouse knee joints injected with methylated bovine serum albumin [59]. Furthermore, ST2^-/- ^mice developed attenuated CIA and reduced *ex vivo *collagen-specific induction of pro-inflammatory cytokines (IL-17, TNFα, and IFNγ), and antibody production [55]. Conversely, treatment with IL-33 exacerbated CIA and elevated production of both pro-inflammatory cytokines and anti-collagen antibodies through a mast cell-dependent pathway. Administration of blocking anti-ST2 antibodies at the onset of CIA also attenuated the severity of disease and reduced joint destruction [56]. This was also associated with reduced IFNγ and IL-17 production. In a model of anti-glucose-6-phosphate isomerase autoantibody-induced arthritis, IL-33 treatment exacerbated disease. Conversely, ST2^-/- ^mice were protected against disease and had reduced expression of articular pro-inflammatory cytokines [62]. The IL-33 effects in this model also appear to be mast cell-dependent as IL-33 failed to increase the severity of the disease in mast cell-deficient mice, and mast cells from wild-type, but not ST2^-/- ^mice restored the ability of ST2^-/- ^recipients to respond. IL-33 has also been shown to chemoattract neutrophils to a knee joint injected with methylated bovine serum albumin [59].

Various rheumatological diseases can have effects on bone including erosion (e.g. RA) and ossification and the formation of new bone (e.g., ankylosing spondylitis and OA). Recently, the role of IL-33 in bone metabolism and remodeling has been studied with conflicting results. Bone structure and metabolism are determined by the formation and activity of osteoclasts and osteoblasts. Mun and co-workers showed that IL-33 can stimulate the formation of multi-nuclear osteoclasts from monocytes, and enhanced expression of osteoclast differentiation factors including TRAF6, nuclear factor of activated T cells cytoplasmic 1, c-Fos, c-Src, cathepsin K, and calcitonin receptor [63]. However, in contrast two other studies have shown that IL-33 completely abolished the generation of multinucleated osteoclasts [64] or had no direct effect [65,66].

IL-33 also appears to have direct effects on osteoblast cells. IL-33 expression increases during osteoblast differentiation, and that while ST2^-/- ^mice displayed normal bone formation they had increased bone resorption, thereby resulting in low trabecular bone mass [64]. Furthermore, IL-33 mRNA levels are increased in osteoblasts following treatment with the bone anabolic factors parathyroid hormone or oncostatin M. In addition, IL-33 treatment promoted matrix mineral deposition by osteoblasts *in vitro *[65]. However, a recent study reports conflicting data that while IL-33 mRNA is present in human osteoblasts, ST2L is not constitutively expressed and IL-33 treatment has no effect on these cells [66]. The reasons for these differences in the biology of IL-33 in osteoclasts and osteoblasts are unclear at present but may reflect different cell culture conditions and differentiation protocols used. In summary, IL-33 appears to have pro-inflammatory effects in various rheumatological diseases activating synovial fibroblasts and mast cells within joints.

#### Inflammatory skin disorders

Skin and activated dermal fibroblasts contain a high level of IL-33 mRNA expression compared to other tissues and cell types [3]. IL-33 mRNA and protein is also substantially higher in the skin lesions of patients with atopic dermatitis compared with non-inflamed skin samples [67], and in affected psoriatic skin compared to healthy skin [68,69]. Elevated serum IL-33 levels have also been detected in patients with systemic sclerosis, and levels correlated positively with the extent of skin sclerosis [70]. Furthermore, subcutaneous administration of IL-33 can induce IL-13-dependent fibrosis of skin in murine models [71]. Recently, it was shown that ST2^-/- ^mice exhibited reduced cutaneous inflammatory responses compared to WT mice in a phorbol ester-induced model of skin inflammation [69]. Furthermore, intradermal injections of IL-33 into the ears of mice induced a psoriasis-like inflammatory lesion that was partially dependent on mast cells.

**Figure 2:**
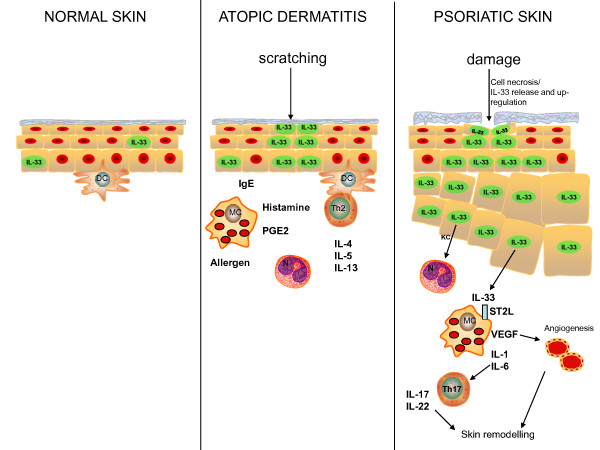
**Schematic representation of the potential pro-inflammatory role of IL-33 in normal skin and in skin inflammation (atopic dermatitis and psoriasis)**. Damage to the skin such as by scratching in response to an allergen and inflammation lead to cell necrosis and release of biologically active IL-33. IL-33 can interact with its receptor ST2L on a number of cell types within the skin, including resident skin cells and infiltrating immune cells. IL-33 may drive dendritic cell (DC) mediated polarization of naïve CD4^+ ^T cells towards a Th2 phenotype and the production of cytokines such as IL-5, IL-10 and IL-13. IL-33 can also potently activate innate immune cells such as mast cells (MC) leading to release of biologically active mediators such as VEGF, histamine and prostaglandin E2 (PGE2). IL-33 can also lead to production of the chemokine KC, thus recruiting neutrophils (N). An increase in Th17 cells and related cytokines IL-17/22 may be driven by IL-33 stimulation of IL-1 and IL-6 production. Furthermore, IL-33 mediated production of VEGF may drive angiogenesis and skin remodeling.

In addition, IL-33 expression was induced in pericytes in an experimental model of wound healing in rat skin [72]. Surprisingly, IL-33 has also been shown to induce cutaneous hypernociception in mice, a phenomenon traditionally associated with Th1 responses [73]. Collectively, these results demonstrate that IL-33 may play a role in various inflammatory skin disorders (Figure 2).

#### Inflammatory bowel disease (IBD)

IBD is a group of chronic inflammatory conditions of the colon and small intestine, including ulcerative colitis (UC) and Crohn's disease, resulting from dysregulated immune responses. Several studies report an upregulation of IL-33 mRNA in human biopsy specimens from untreated or active UC patients compared to controls [72,74-77]. The main sites of UC IL-33 expression were myofibroblasts and epithelial cells. Similarly, ST2 transcripts have been detected in mucosa samples from patients with active UC [74,75]. However, although Carriere and co-workers demonstrated expression of IL-33 in endothelial cells of Crohn's disease intenstine [11], subsequent studies have failed to demonstrate a significant role for IL-33 in Crohn's disease [72,74,76]. Serum IL-33 and sST2 levels were elevated in UC patients compared with controls, while anti-TNF treatment decreased circulating IL-33 and increased sST2, thus favorably altering the ratio of the cytokine with its decoy receptor [74]. However, in other studies serum concentrations of IL-33 were low or did not differ between UC patients and healthy controls [75,78].

Several murine studies highlight a role for IL-33 in innate-type immunity in the gut. Mice treated with IL-33 displayed epithelial hyperplasia and eosinophil/neutrophil infiltration in the colonic mucosa [3]. Furthermore, in a murine model of T-cell independent dextran sodium sulphate (DSS)-induced colitis IL-33^-/- ^mice had enhanced viability, compared to wild-type controls [19]. In a related study macrophage-specific transgenic mice that express a truncated TGF-β receptor II under control of the CD68 promoter (CD68TGF-βDNRII) and subjected to the DSS model of colitis display an impaired ability to resolve colitic inflammation but also an increase in IL-33^+ ^macrophages compared to controls [79]. In addition, IL-33 mRNA is upregulated in the ilea and correlates with disease severity in a murine model of Th1/Th2-mediated enteritis, and induced IL-17 production from mesenteric lymph node cells stimulated *ex vivo *[74]. In summary, the IL-33/ST2 pathway may be an important regulator of UC, but be of less importance in Crohn's disease.

#### Central nervous system (CNS) inflammation

Basal IL-33 mRNA levels are extremely high in the brain and spinal cord [3], and are elevated under conditions such as experimental subarachnoid hemorrhage [1]. Furthermore, expression of IL-33 in glial and astrocyte cultures is increased by Toll-like receptor ligands [80]. Treatment with IL-33 induces proliferation of microglia and enhances production of pro-inflammatory cytokines, such as IL-1β and TNFα, as well as the anti-inflammatory cytokine IL-10 [81]. It also enhances chemokines and nitric oxide production and phagocytosis by microglia. In mice, IL-33 levels and activity were increased in brains infected with the neurotropic virus Theiler's murine encephalomyelitis virus [80]. Finally, a transcriptional analysis of brain tissue from patients with Alzheimer's disease revealed that IL-33 expression was decreased compared to control tissues [82]. This study also demonstrated that 3 polymorphisms within the IL-33 gene resulting in a protective haplotype were associated with risk of Alzheimer's disease [82]. This data is supported by a study in Chinese population with evidence that genetic variants of IL-33 affect susceptibility to Alzheimer's disease [83]. Furthermore, cell-based assays demonstrate that IL-33 can decrease secretion of β-amyloid peptides [82]. Thus, IL-33 may have a role in regulating pathophysiology and inflammatory responses in the CNS.

### Cancer

Although early reports document the expression of ST2 on leukaemic cell lines and on T cell lymphomas of patients [84,85], very few studies have addressed the role of IL-33/ST2 signaling on anti-tumor immune responses, tumor growth and/or metastasis. However, a recent study demonstrated that ST2^-/- ^mice with mammary tumors have attenuated tumor growth and metastasis, with increased circulating levels of pro-inflammatory cytokines and activated NK and CD8^+ ^T cells [86]. Furthermore, IL-33 induces proliferation, migration, and morphologic differentiation of endothelial cells, consistent with an effect on angiogenesis [87]. In addition, IL-33 expression is present in endothelial cells of healthy organs but is strikingly absent from those in tumors [88]. Therefore, IL-33 may be an important mediator in tumor escape from immune control and in tumor angiogenesis and thus warrants further investigation.

#### Cardiovascular (CV) disease

IL-33 was initially found in the nucleus of the high endothelial venules (HEV) of secondary lymphoid tissues [2]. More recently, IL-33 expression has been reported in coronary artery smooth muscle cells [3], coronary artery endothelium [89], non-HEV endothelial cells [88,90], adipocytes [66,91], and in cardiac fibroblasts suggesting that IL-33 may play a role in various CV disorders [92].

##### sST2 as a CV biomarker

This concept is supported by the clinical finding that the IL-33 decoy receptor sST2 was elevated in serum early after acute myocardial infarction (AMI), and correlated with creatine kinase and inversely correlated with left ventricular ejection fraction [93]. Since this primary observation several studies have since demonstrated the prognostic value of measuring serum sST2 in various CV diseases, showing that high baseline levels of sST2 were a significant predictor of CV mortality and heart failure (HF) (Table 1). Taken together, these studies indicate that sST2 has the potential to be a predictive CV biomarker in patients with AMI, HF and dyspnea. Thus far, serum or plasma IL-33 has not been measured in CV disease. While levels are elevated in atopy [67], and some rheumatological diseases [57,58], the levels in CV disease are likely to be low (possibly due to elevated sST2 levels) and difficult to measure with currently available assays. However, recent studies have highlighted the development of multiplex assays to measure low abundance IL-33 in serum or plasma and warrant further investigation in the context of CV disease [94]. In summary, sST2 shows promise as a biomarker predictive of mortality in several CV disorders.

**Table 1:** Studies examining sST2 in serum/plasma of patients with CV disease

Disease	Result	Ref.
AMI	• sST2 levels were increased in the serum of patients 1 day after AMI.	[93]
	• ST2 levels predicted subsequent mortality and HF in patients admitted with AMI (TIMI, STEMI & CLARITY-TIMI trials).	[103,104]
	• sST2 levels predicted adverse left ventricular functional recovery and remodeling post-AMI.	[105]

Acute chest pain	• Measurement of sST2 was of no prognostic value in the prediction of AMI, acute coronary syndromes or 30-day events in patients presenting to the emergency department with chest pain.	[106]

HF	• PRAISE-2 HF trial and showed that the change in sST2 levels was an independent predictor of subsequent mortality or transplantation in patients with severe chronic HF.	[107]
	• Increased plasma concentrations of sST2 are predictive for 1-year mortality in patients with acute destabilized HF.	[108]
	• sST2 levels correlated with the severity of HF and left ventricular ejection fraction.	[109]
	• Serial sampling of sST2 demonstrated that the % change in sST2 concentrations during acute HF treatment is predictive of 90-day mortality.	[110]
	• Elevated sST2 concentrations are predictive of sudden cardiac death in patients with chronic HF.	[111]
	• Pleural fluid sST2 levels were not helpful for diagnosing effusions due to HF.	[112]
	• sST2 levels were lower in decompensated HF patients who did not have a sudden cardiac event.	[113]
	• sST2 levels were greater in patients with systolic HF than in those with acutely decompensated HF with preserved ejection fraction.	[114]
	• Chronic HF patients whose sST2 levels were in the highest had a markedly increased risk of adverse outcomes compared with the lowest tertile.	[115]

Cardiac Surgery	• Cardiac surgery patients undergoing coronary artery bypass grafting with cardiopulmonary bypass demonstrate a significant rise in sST2 levels 24 hours after surgery.	[116,117]

Outpatient study	• In an outpatient study sST2 levels also reflected right-side heart size and function and were an independent predictor of 1-year mortality in outpatients referred for echocardiograms.	[118]

Dyspnea	• sST2 concentration strongly predicted death at 1 year in dyspneic patients.	[119-122]
	• sST2 concentrations are associated with cardiac abnormalities on echocardiography, a more decompensated hemodynamic profile and are associated with long-term mortality in dyspneic patients.	[123]

AMI - Acute Myocardial Infarction; HF - Heart Failure

##### Cardiac fibrosis and hypertrophy

Studies in animal models suggest that sST2 is more than just a marker in CV disease and implicate IL-33/ST2 signaling as an important protective pathway in various CV diseases. In a model of pressure overload IL-33 treatment reduced cardiac hypertrophy and fibrosis, and improved survival following transverse aortic constriction in wild-type but not ST2^-/- ^mice [92]. Furthermore, sST2 blocked the anti-hypertrophic effects of IL-33, indicating that sST2 functions in the myocardium as a soluble decoy receptor of IL-33. IL-33 can also reduce cardiomyocyte apoptosis, decrease infarct and fibrosis, and improve ventricular function *in vivo *via suppression of caspase-3 activity and increased expression of the 'inhibitor of apoptosis' family of proteins [95]. The protective effects of IL-33 may be limited by the neurohormonal factor endothelin-1, which increased expression of sST2 and inhibited IL-33 signaling through p38 MAP Kinase [96].

##### Atherosclerosis

During atherosclerosis immune cells such as monocytes, T cells and mast cells infiltrate plaques within the intima of the arterial wall [97]. The disease appears to be driven by a Th1 immune response with cytokines such as IL-12 and IFNγ inducing pathogenesis [98,99]. Thus, it was hypothesized that IL-33 may have protective effects during atherosclerosis by inducing a Th1-to-Th2 switch of immune responses. In fact, treatment of ApoE^-/- ^mice with IL-33 significantly reduced atherosclerotic lesion size in the aortic sinus and reduced plaque F4/80^+ ^macrophage and CD3^+ ^T cell content [26]. IL-33 treatment increased levels of the Th2 cytokines IL-4, IL-5, and IL-13 but decreased levels of the Th1 cytokine IFNγ in serum and lymph node cells. Furthermore, IL-33-treated ApoE^-/- ^mice also produced significantly elevated levels of protective anti-oxidized low-density lipoprotein (ox-LDL) IgM antibodies. Conversely, mice treated with intraperitoneal injections of sST2 developed significantly larger atherosclerotic plaques and enhanced IFNγ levels. Thus far, atherosclerosis development has not been studied in ApoE^-/- ^or LDLR^-/- ^mice also deficient in genes encoding either IL-33 or ST2 and these studies are required in order to examine the endogenous role of IL-33. Cell-based experiments have also shown that IL-33 has potent effects on macrophage-derived foam cell function *in vitro *providing further evidence for anti-atherosclerotic effects of IL-33 [100]. Taken together these results indicate that IL-33/ST2 signaling may play a protective role in atherosclerosis.

##### Obesity and type 2 diabetes

**Figure 3:**
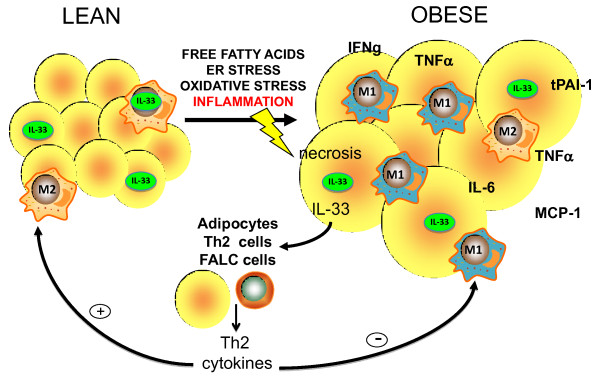
**Schematic representation of the potential ant-inflammatory role of IL-33 in adipose tissue inflammation**. Tissue damage caused by factors such as high free fatty acids, ER stress, oxidative stress, and inflammation can lead to necrosis of cells and release of biologically active IL-33. This can interact with its receptor ST2L on a number of cell types within adipose tissue (adipocytes themselves, CD4^+ ^Th2 cells and Fat-Associated Lymphoid Cluster (FALC) cells) leading to the production of protective Th2 cytokines (e.g. IL-5, IL-10 and IL-13). IL-33 can polarize macrophages towards an alternatively activated (M2) phenotype and reduce lipid uptake in adipocytes and macrophages via the down-regulation of several metabolic genes.

Recently, expression of IL-33 and ST2 was reported in adipocytes and adipose tissues [66,91]. Subsequently it was shown that treatment of adipocyte cultures *in vitro *with IL-33 induced the production of Th2 cytokines (IL-5 and IL-13), reduced lipid storage and decreased the expression of several genes associated with lipid metabolism and adipogenesis (e.g. C/EBPα, SREBP-1c, LXRα, LXRβ, and PPARγ) [101]. Furthermore, treatment of genetically obese diabetic (*ob/ob*) mice with IL-33 led to protective metabolic effects with reduced adiposity, reduced fasting glucose and improved glucose and insulin tolerance [101]. Conversely, ST2^-/- ^mice fed high fat diet for 6 months had increased body weight and fat mass, impaired insulin secretion and glucose regulation compared to wild-type controls. The protective effects of IL-33 on adipose tissue appear to be mediated via an increased production of Th2 cytokines and a switching of macrophage polarization from an M1 to an M2 phenotype (Figure 3). More recently, a newly identified population of cells expressing ST2 were found in adipose named natural helper cells or fat-associated lymphoid cluster (FALC) cells that produce large amounts of Th2 cytokines in response to IL-33 [102], but the direct role of these cells in obesity is still unclear.

## Conclusions

IL-33 appears to be a crucial cytokine for Th2-mediated host defense and plays a central role in controlling immune responses in barrier tissues such as skin and intestine. It is able to activate cells of both the innate and adaptive immune system, and depending on the disease can either promote the resolution of inflammation or drive disease pathology. Manipulation of the IL-33/ST2 pathway therefore represents a promising new therapeutic strategy for treating or preventing various inflammatory disorders. However, many questions regarding the fundamental biology of IL-33 remain to be solved, including its nuclear effects and processing and release of IL-33 from cells. Furthermore, given the wide variety of cellular responses regulated by IL-33 and ST2, and in particular the cardio-protective effects of IL-33, this should be approached with caution.

## List of abbreviations

AMI: Acute myocardial infarction; CIA: Collagen-induced arthritis; CNS: Central nervous system; CV: Cardiovascular; HEV: High endothelial venules; HF: Heart failure; IL: Interleukin; IL:1RAcP- IL:1R accessory protein; MAPK: Mitogen-activated protein kinase; OA: Osteoarthritis; PsA: Psoriatic arthritis; RA: Rheumatoid arthritis; SIGIRR: Single Ig IL:1R-related molecule; SLE: Systemic lupus erythematosus; sST2: Soluble ST2; Th: T helper; TIR: Toll-interleukin-1 receptor; TLR: Toll-like receptor; UC: Ulcerative colitis

## Competing interests

The authors declare that they have no competing interests.
